# Environmental Influences on Dietary Intake of Children and Adolescents

**DOI:** 10.3390/nu12040922

**Published:** 2020-03-27

**Authors:** Jessica S. Gubbels

**Affiliations:** Department of Health Promotion, NUTRIM School of Nutrition and Translational Research in Metabolism, Maastricht University, NL-6200 MD, Maastricht, The Netherlands; jessica.gubbels@maastrichtuniversity.nl

## Introduction

Childhood is a crucial period for establishing lifelong healthy nutritional habits [1]. The environment can have an important influence on these habits [2]. According to the ANGELO framework [3], the environment can be operationalized through distinguishing between four environmental types: The physical environment (what is available), the social environment (what are the attitudes/beliefs of important others), the political environment (what are the rules), and the economic environment (what are the costs) [3]. The studies described in the current special issue cover these various types of environmental influences within different settings, including home and school, on children and adolescents’ dietary intake.

Several studies in the special issue focused on environmental influences in the home setting. Parents play an important role in forming their children’s dietary habits and are important gatekeepers for children’s behavior [4,5]. Gibson and colleagues [6] examined parental influences on snacking in preschoolers in a large cross-European sample. Parents’ own snacking behavior and rules about snacking were significantly associated with the intake of their children, but parents’ educational level and nutritional knowledge were also important predictors. Interestingly, the parental influences were different for healthy and unhealthy snacking [6]. In an older sample, Van Lippevelde and colleagues [7] showed that health-promoting parenting practices were associated with reduced sugar sweetened beverage intake and increased healthy snack intake, while health-reducing practices were associated with increased unhealthy snack intake. Van Lippevelde and colleagues [7] also examined whether the parenting practices moderated the positive relationship between adolescents’ reward sensitivity and unhealthy dietary intake, but this was not the case. Interestingly, they did find indications of such an interaction when using the parent-reported instead of the adolescent-reported parenting practices as a moderator [7]. This stresses the importance of carefully considering our methodology to assess environmental influences, also taking the child’s perspective of the environment into account [8]. In addition, Hermans and colleagues [9] showed that parental influence on adolescents’ dietary intake even reaches beyond the home setting: Support from mothers was positively associated with adolescents bringing healthy products to school, and negatively associated with their purchase of sweet snacks in and around school [9].

Tani, Fujiwara, Doi, and Isumi [10] examined the association between home cooking and childhood obesity in a large sample of primary school children in Japan. Children living in households with a low frequency of home cooking were more than twice as likely to be obese as those in a household with high cooking frequency were. This association was partially mediated by children’s diet (vegetable, breakfast, and snack intake), suggesting that home cooking is associated with healthy intake, which, in turn, decreases the risk for obesity [10]. Home cooking and family meals are important indicators of family functioning [11], and can thus be regarded an important target for future obesity prevention interventions. Transference of cooking skills from parents to their children could be a key aspect in increasing home cooking. Lavelle and colleagues [12] examined such transference of cooking skills in a qualitative study among Irish mothers. Although mothers expressed a desire for teaching their children to cook, various barriers to actually involving children in cooking were identified. These barriers included children’s lack of interest in cooking, clingy behavior and messiness in the kitchen, and pickiness with regard to food. This points out that parents may need some help in dealing with practical barriers to involving their children in the kitchen, as well to motivate their children to actually get involved [12].

In addition to parental influences, increasing attention is being paid to the influence of the broader family system on children’s dietary intake [13]. In the current special issue, Verjans-Janssen and colleagues [14] examined the associations between the Family Nutrition Climate—i.e., the family’s shared perceptions and cognitions regarding healthy nutrition [15]—and dietary intake of primary school children. Several subscales, as well as the total Family Nutrition Scale, were positively associated with healthy intake behaviors, including increased fruit, vegetable, and water intake, and decreased soft drink and sweets intake [14]. This underlines the importance of the family-level influences in addition to parental influences.

Furthermore, parents often share responsibility for caring for their child with (pre)schools and childcare facilities. Whereas the majority of young children in OECD (Organisation for Economic Co-operation and Development)-countries attend some form of preschool or organized childcare [16], almost all older children and adolescents attend some type of education [17]. In the current special issue, various papers addressed the influence of care and educational facilities.

With regards the youngest children, Korkalo and colleagues [18] showed that preschool meals contribute to over half of weekday energy intake in young Finnish children. This shows the important influence preschools can have on children’s diets. Preschool meals were relatively healthy, being high in fibers, fish, unsaturated fats, and several vitamins, and low in added sugar. However, fruit consumption at preschool was low and salt intake was relatively high [18]. These findings point out the specific intake behaviors that need to be addressed in future interventions. In another study from Finland, Lehto and colleagues [19] examined the association of various facilitators and barriers at preschool, with children’s fruit, vegetable, and fiber intake in those preschools. One of the main predictors of healthy intake was the presence of food policies [19]. Policies are crucial for the longer-term maintenance of potential intervention effects [20], preventing that established environmental changes slowly dilute and eventually diminish over time. Surprisingly, the study of Lehto et al. [19] did not find any influence of center cooking facilities, resources, or staff education on children’s intake, which might imply that a lack of resources and facilities does not necessarily hinder healthy dietary intake. Benjamin Neelon and colleagues [21] also examined barriers to the implementation of intervention in childcare settings, though specifically for fruit and vegetable gardens. Fruit and vegetable garden projects have been previously shown to increase children’s intake of fruit and vegetables (e.g., [22], but to date few interventions in Early Care and Education settings have included gardening [23]). Benjamin Neelon and colleagues [21] showed that although the majority (81%) of the English childcare settings was interested in implementing gardens, various practical barriers hindered actual implementation, including lack of space, expertise and time. This is in line with the findings of Holley and colleagues [24], who identified various barriers and challenges for free food provision at holiday clubs to tackle children’s hunger. Although reported effects did not only include tackling of hunger, but also creating positive food experiences and promoting social interactions and positive behavior in general, the reported challenges, including resources constraints, hindered implementation [24]. This indicates that a tailored approach is necessary when implementing intervention approaches, taking into account intermediaries’ needs and barriers, as well as their strengths [25].

Moving on to primary schools, Bartelink and colleagues examined effects of an integrated intervention on children’s diet and physical activity in a large quasi-experimental study in the Netherlands [26]. They found the intervention to have favorable effects on children’s diet and physical activity. However, the effects on diet were only present when the full intervention was implemented: Schools that skipped the free healthy lunch that was part of the intervention only found effects on physical activity. In addition, the intervention showed less favorable effects on younger children and children with a low socioeconomic status (SES). These findings of Bartelink and colleagues [26] are in line with an ecological systems view of environmental influences on behavior [27,28,29], which states that environmental influences on diet are context-specific (like children’s SES in the study by Bartelink et al.) as well as person-specific (in line with the diminished effects depending on children’s age in the study). Hence, determinants of behavior cannot be viewed in isolation. They influence each other, and it is their combined influence that determines behavior [30]. This stresses the importance of a broad, integrative approach in interventions. The findings of Kiss and colleagues [31] underline this. In their analysis of the reform of the school catering system in Hungary, they showed that changed regulations (i.e., the political environment) are not necessarily translated into action. All involved sectors need to be on board, and interaction and dialogue between stakeholders needs to be facilitated. Furthermore, they stressed that there is no universal solution fitting all settings and all children [31], in line with the different effects depending on contextual and person-related factors reported by Bartelink et al. [26].

Diet is also a concern within secondary schools. Garrido-Fernández and colleagues [32] showed that adolescents were more likely to consume unhealthy snacks when they attended a school with a cafeteria. Hence, while school cafeteria might have large potential to improve children’s diet and prevent overweight [33], this potential is often not utilized in practice [32]. In addition to the physical environment at secondary schools, the social environment is also very important. Trigueros and colleagues [34,35] showed that teaching style during Physical Education (PE) classes was associated with Portuguese adolescents’ motivation, which, in turn, was related to their diet and physical activity. Their findings support the Self Determination Theory [36]: Satisfaction of the basic human psychological needs (autonomy, competence, and relatedness) was crucial for the adolescent’s motivation toward PE classes. Students that were highly motivated consumed more healthy foods and less unhealthy foods and were more active. As such, Trigueros et al. [34,35] provide us with insight into some of the cognitive factors that precede behavioral decisions regarding dietary intake and how the environment can support these cognitive processes. The results can therefore be used by interventions addressing PE classes and teaching styles.

Moving from the school setting to the surrounding neighborhood environment, Díez and colleagues [37] examined socioeconomic inequalities in the food environment around schools in Spain. They showed that 95% of the schools were surrounded by unhealthy food retailers within a short range, with a median of 17 unhealthy food outlets per school. A worrying addition to this was that unhealthy food retailers were both closer and higher in number for schools in low-SES neighborhoods [37], indicating that children from a lower SES background are growing up in a less healthy food environment. Dhillon and colleagues [38] painted an equally problematic picture regarding the food environment of college students living on a food-desert campus in the United States. Performing a qualitative focus group study, they showed that there was a lack of adequate, acceptable, affordable, and accessible food within students’ environment. Healthy foods, such as fruit, were too expensive and often not available at campus, and it was very difficult to get food from outside the campus area [38]. Based on the studies of Díez [37] and Dhillon [38], we can conclude that both the overwhelming offer of unhealthy foods and the lack of healthy foods around educational facilities are very problematic.

Within food outlets, the study by Elliott [39] showed that 88% of Canadian child-targeted products was not suitable for marketing to children. Interestingly, the percentage of children’s products that was unsuitable was stable between 2009 and 2017. At both time points, over 70% of children’s food products was too high in sugar. However, a very large increase in nutrition claims was visible in the observed time period. Also, the use of several marketing techniques aimed at children (e.g., the use of cartoon images and child fonts) increased, while the use of other approaches decreased (e.g., the use of games on the package). The study by Elliott [39] showed the significant pressure to eat unhealthy foods that is exerted by marketing. In line with this, the findings of Czoli et al. [40] showed that although overall exposure to food advertisements among Canadian children decreased, advertisements were dominated by fast food and sugary drinks. More strict regulation of marketing directed at children and adolescent is therefore urgently needed, which should be directed at the different settings and media that youngsters encounter.

In conclusion, the papers in the current issue underline the importance of the environment in influencing children’s and adolescents’ dietary intake across different settings and types of environments. In addition, the papers identified some crucial barriers and facilitators for the implementation of environmental changes to enable a healthy diet for young children. The special issue therefore provides some important directions for both future research and practice.
